# Supplementary material for: Eukaryotic Pif1 helicase unwinds G-quadruplex and dsDNA using a conserved wedge
Source: Nat Commun. 2024 Jul 19;15:6104. doi: 10.1038/s41467-024-50575-8 (PMC11275212; doi:10.1038/s41467-024-50575-8)
Supplement: Supplementary file 1 — Supplementary Information [file 41467_2024_50575_MOESM1_ESM.pdf]

Supplementary Information

Eukaryotic Pif1 helicase unwinds G-quadruplex and dsDNA using a conserved wedge

Zebin Hong[1,#], Alicia K. Byrd[2#*], Jun Gao[2], Poulomi Das[1], Vanessa Qianmin Tan[1], Emory G. Malone[2], Bertha Osei[2], John C. Marecki[2], Reine U. Protacio[2], Wayne P. Wahls[2], Kevin D. Raney[2*] and Haiwei Song[1,*]

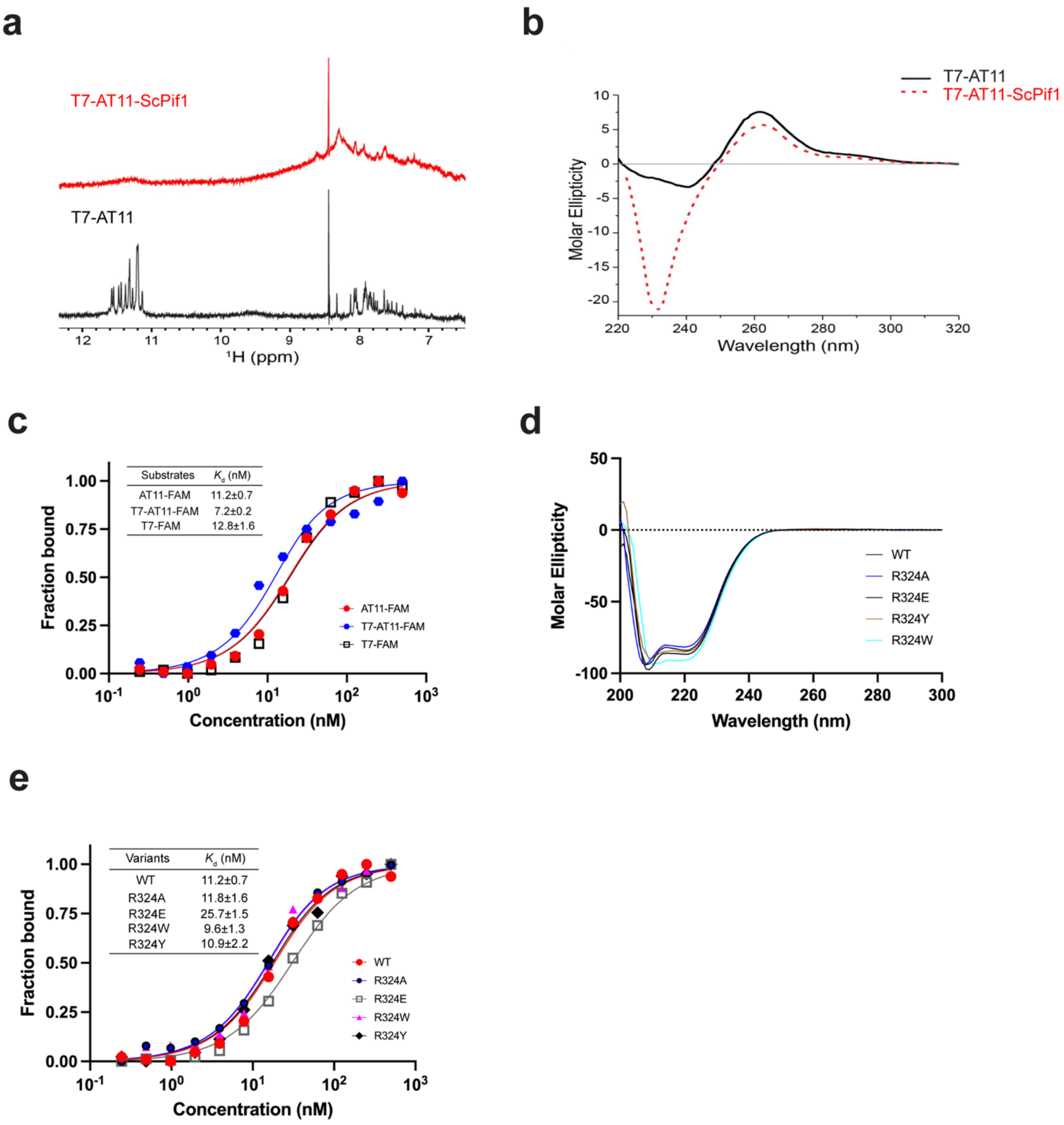

Supplementary Figure 1. Identification of AT11 as the G4 DNA substrate of ScPif1

(**a**) 1D ^{1}H NMR spectra and (**b**) CD spectra demonstrating the complex formation between ScPif1 and AT11. **(c)** The binding of wild type ScPif1 to the DNA substrates examined by fluorescence anisotropy assay. **(d)** CD analysis of wild type ScPif1 and its variants. **(e)** The binding of AT11-FAM to wild type ScPif1 and its variants examined by fluorescence anisotropy assay. Source quantification data for **(c)** and **(e)** are provided as a Source Data file.

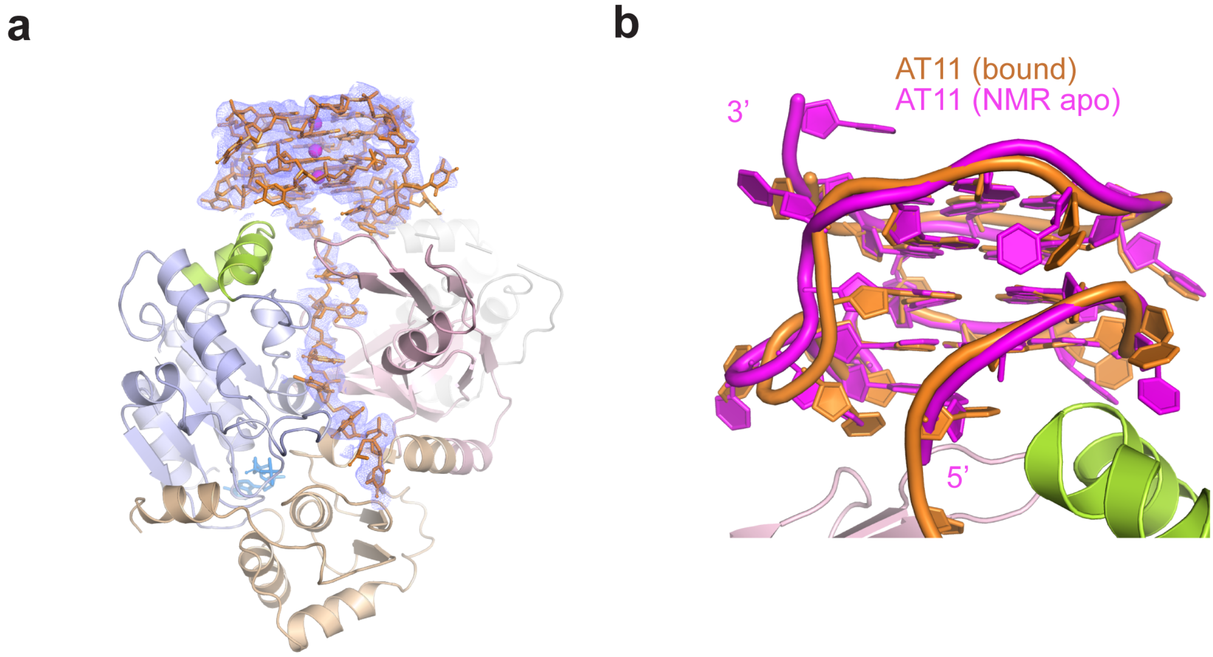

Supplementary Figure 2. AT11 G4 DNA in the structure of ScPif1-G4

(**a**) Structure of ScPif1 with bound AT11 G4 DNA. The cartoon representation of ScPif1 is color-coded as in Figure 1. $2F_O$- F_C density contoured at 1.2 σ around the AT11 DNA with a 5’ ssDNA loading site is shown as blue mesh with G4 DNA in sticks. (**b**) The figure shows a structural superposition of the cartoon representations of the AT11 NMR structure and the ScPif1 bound AT11 structure, colored in magenta and orange, respectively.

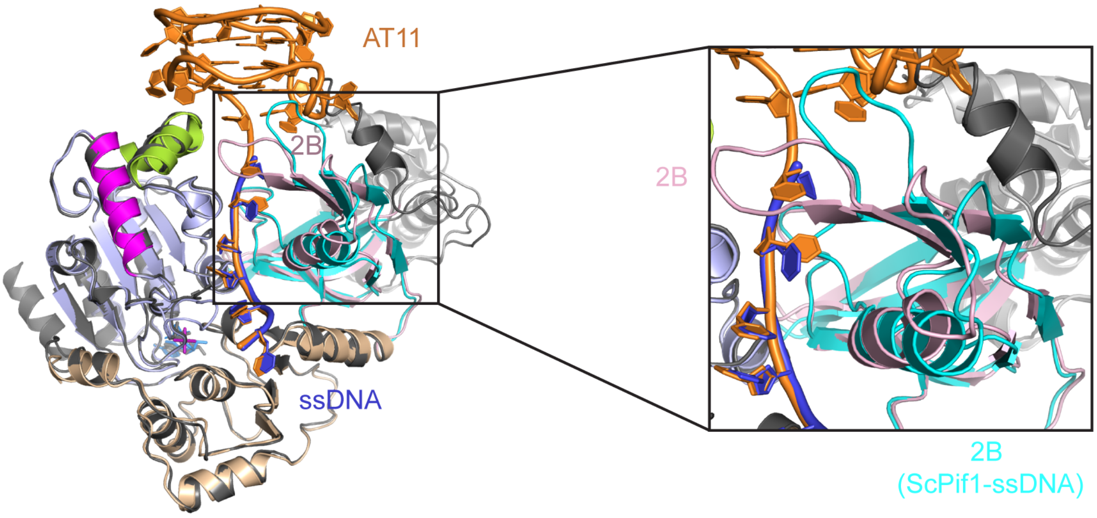

Supplementary Figure 3. Structural superposition of ScPif1-G4 with ScPif1-ssDNA. ScPif1-G4 is color-coded as in Figure 1 while ScPif1 and ssDNA in the ScPif1-ssDNA structure (PDB code: 5O6B) are shown in dark gray and blue, respectively. The 2B domain within ScPif1-ssDNA is shown in cyan.

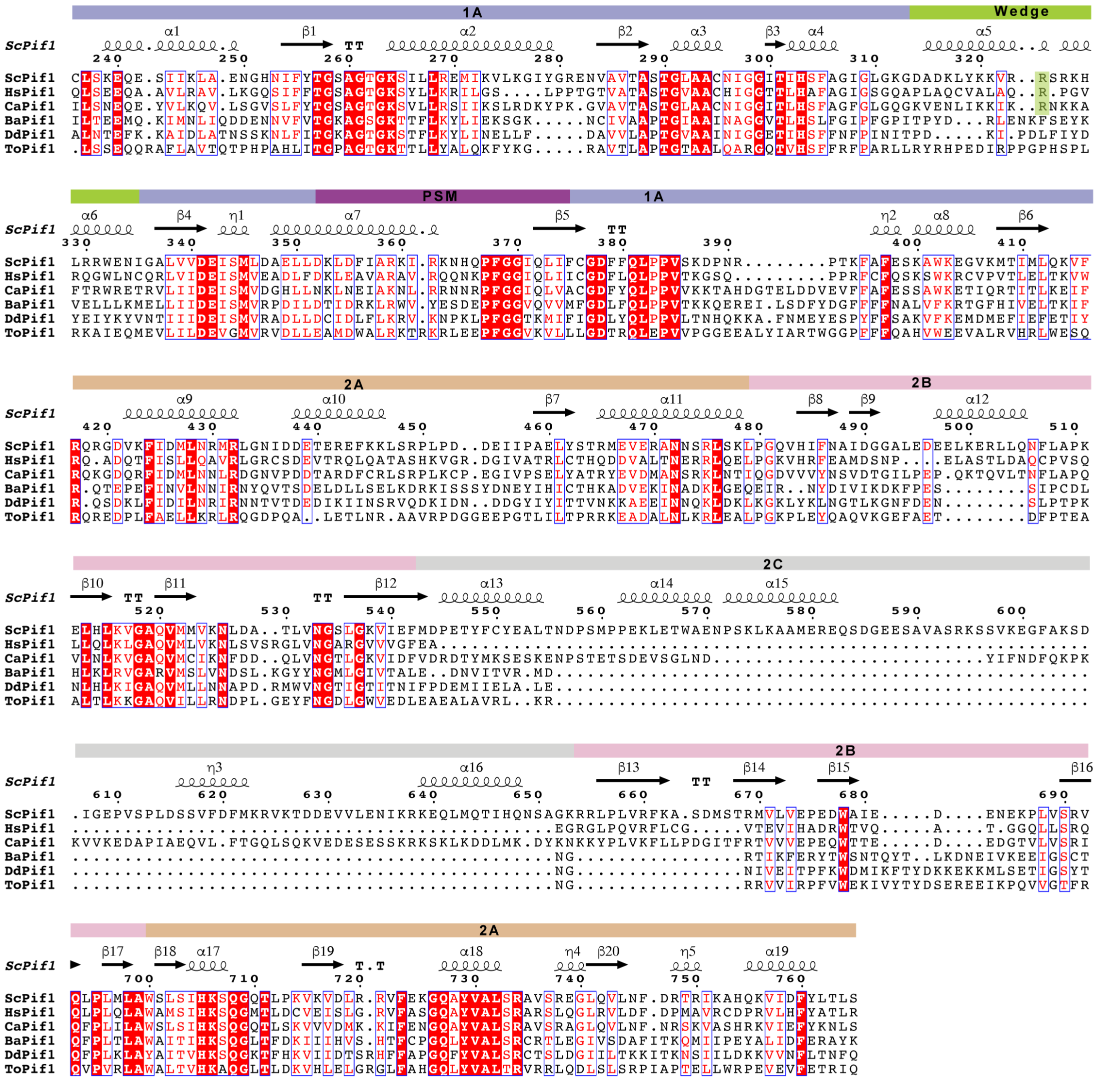

Supplementary Figure 4. Sequence alignment of Pif1 orthologs

Sequences of Pif1 from *Saccharomyces cerevisiae* (Sc), *Homo sapiens* (Hs), *Candida albicans* (Ca), *Bacteroides sp.* (Ba), *Deferribacter desulfuricans* (Dd) and *Thermus oshimai* (To) were aligned with Clustal Omega multiple sequence alignment and depicted using ESPript3[61]. The secondary structural elements of ScPif1 are marked on top of the sequence alignment and color-coded by domain or subdomain as in Figure 1. The key Arg residue in the wedge region is highlighted in limon color.

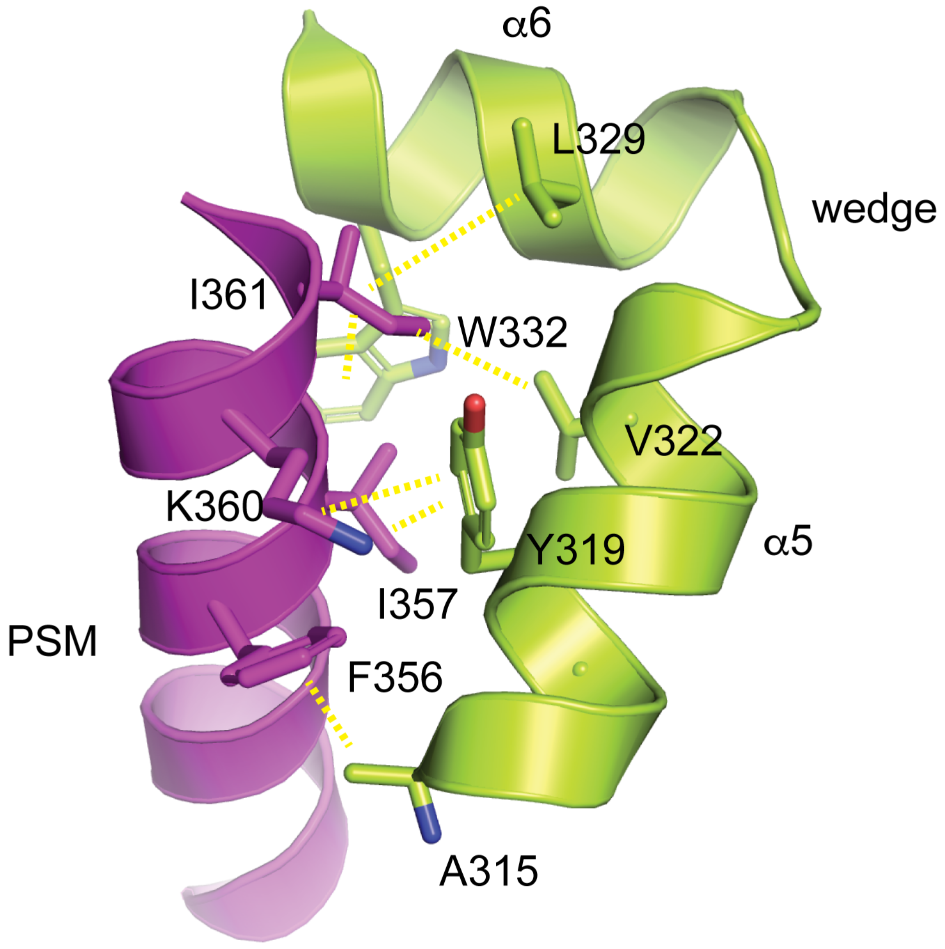

Supplementary Figure 5. Interactions between the wedge and PSM.
Residues involved in the wedge/PSM interaction are shown in sticks color-coded as in Figure 1. The dashed yellow lines show the contacts between interacting residues.

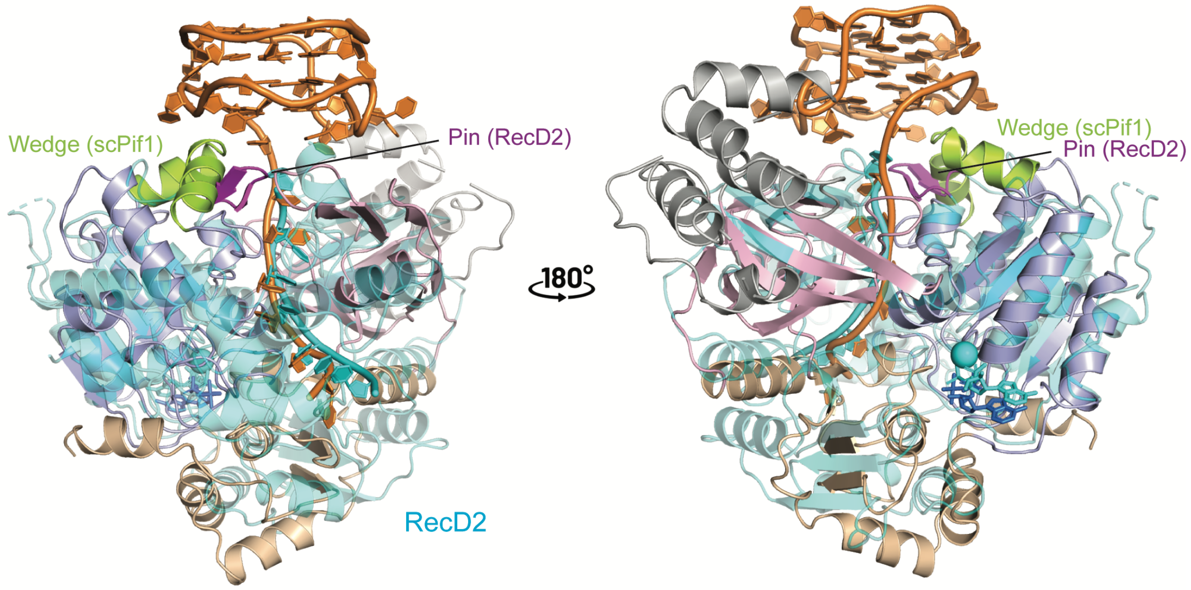

Supplementary Figure 6. Structural comparison of ScPif1-G4 with RecD2

The figure shows the cartoon representations of ScPif1-G4 and RecD2 (PDB code: 3GPL) with bound ssDNA. ScPif1-G4 is color-coded as in Figure 1 with the wedge region critical to DNA unwinding shown in green while RecD2 is colored in cyan with the Pin region critical for dsDNA unwinding highlighted in magenta.

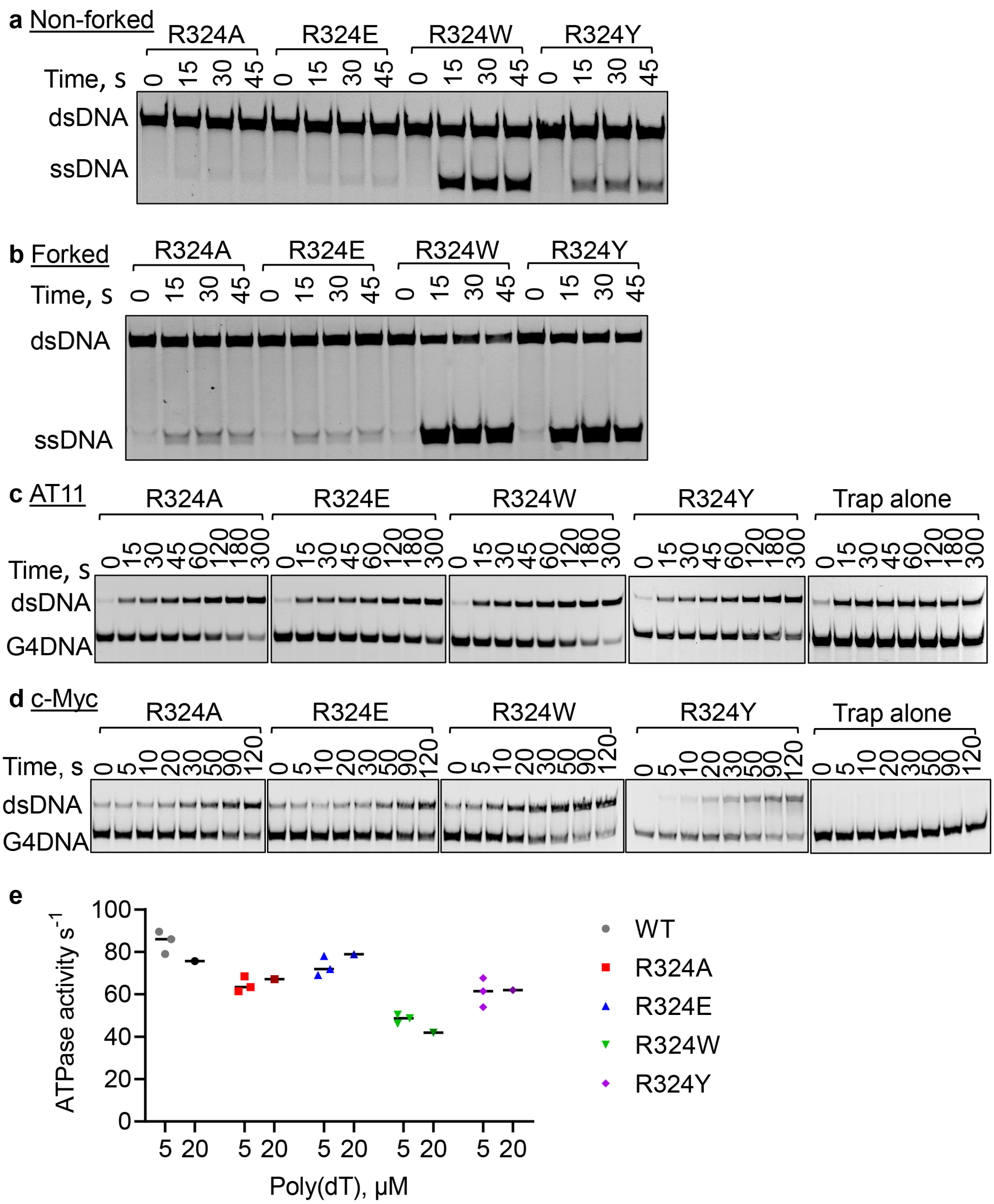

Supplementary Figure 7. R324 is involved in unwinding duplex DNA and unfolding G4DNA but only moderately affects ATPase activity.

Gels show unwinding of a non-forked duplex (**a**), unwinding of a forked duplex (**b**), unfolding of a AT11 (**c**), and unfolding of c-Myc (**d**) by ScPif1 variants. (**e**) Rates of ATP hydrolysis by ScPif1 variants stimulated by a saturating concentration of poly(dT) are plotted. Experiments were performed in triplicate at 5 μM poly(dT), and a single replicate was performed at 20 μM poly(dT). Source gels and quantification data are provided as a Source Data file.

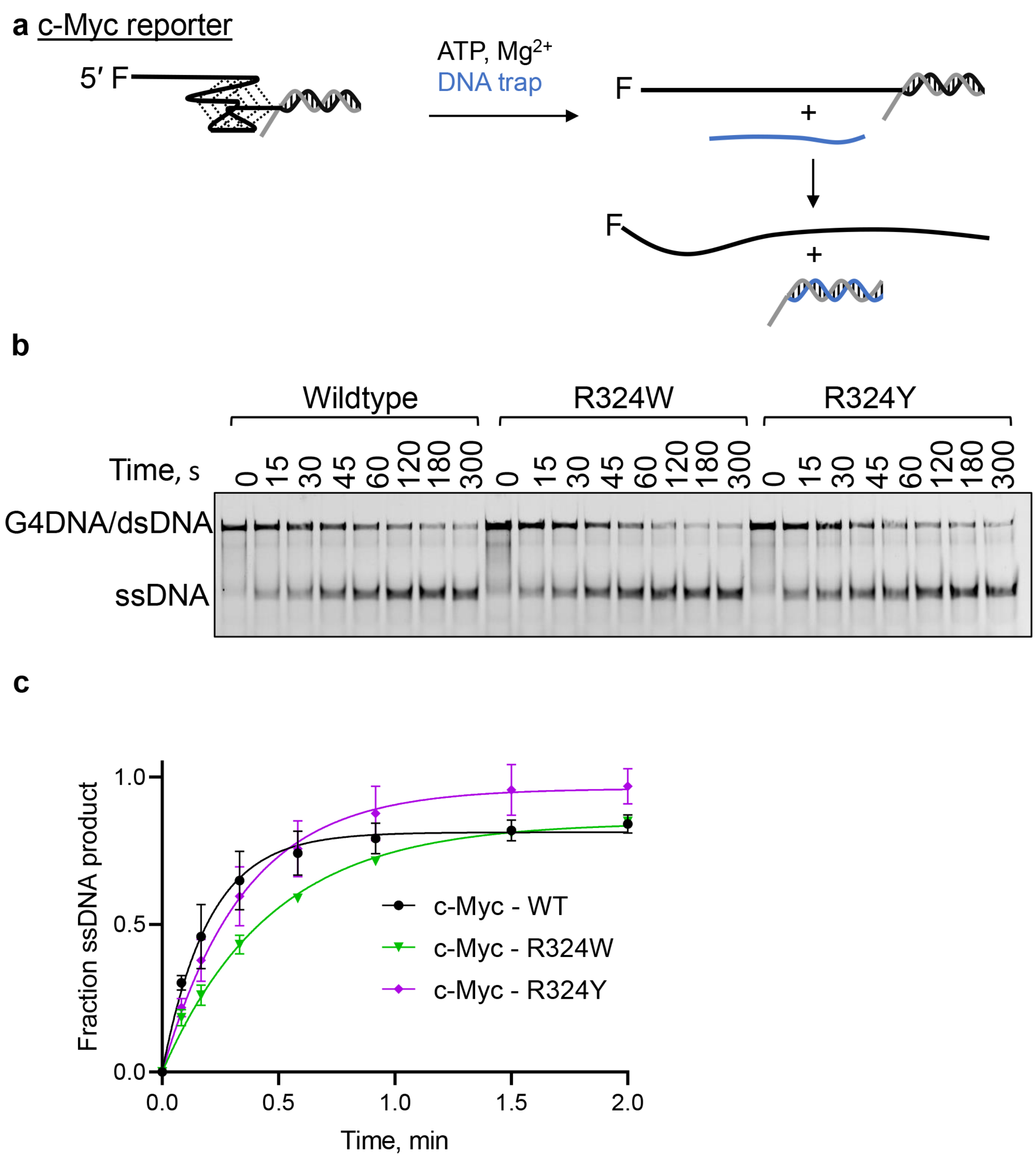

Supplementary Figure 8. R324W and R324Y unfold G4DNA similarly to wildtype ScPif1. (**a**) Illustration of a reporter assay for G4DNA unfolding. Rapid unwinding of the duplex DNA after G4DNA unfolding allows duplex DNA unwinding to serve as a reporter for G4DNA unfolding. (**b**) Unfolding of c-Myc G4DNA by wildtype, R324W, and R324Y Pif1 was measured using a reporter assay. (**c**) Unfolding of c-Myc G4DNA was fit with a single exponential function. Rate constants were 0.083 ± 0.022 s^{-1}, 0.036 s^{-1}, and 0.048 ± 0.007 s^{-1} for unfolding by wildtype, R324W, and R324Y ScPif1, respectively. The amplitudes of product formation were 0.81 ± 0.03, 0.845, and 0.960 ± 0.07 for unfolding by wildtype, R324W, and R324Y ScPif1, respectively. Results are average and standard deviation of triplicate experiments except R324W is duplicate experiments. Source gels and quantification data are provided as a Source Data file.

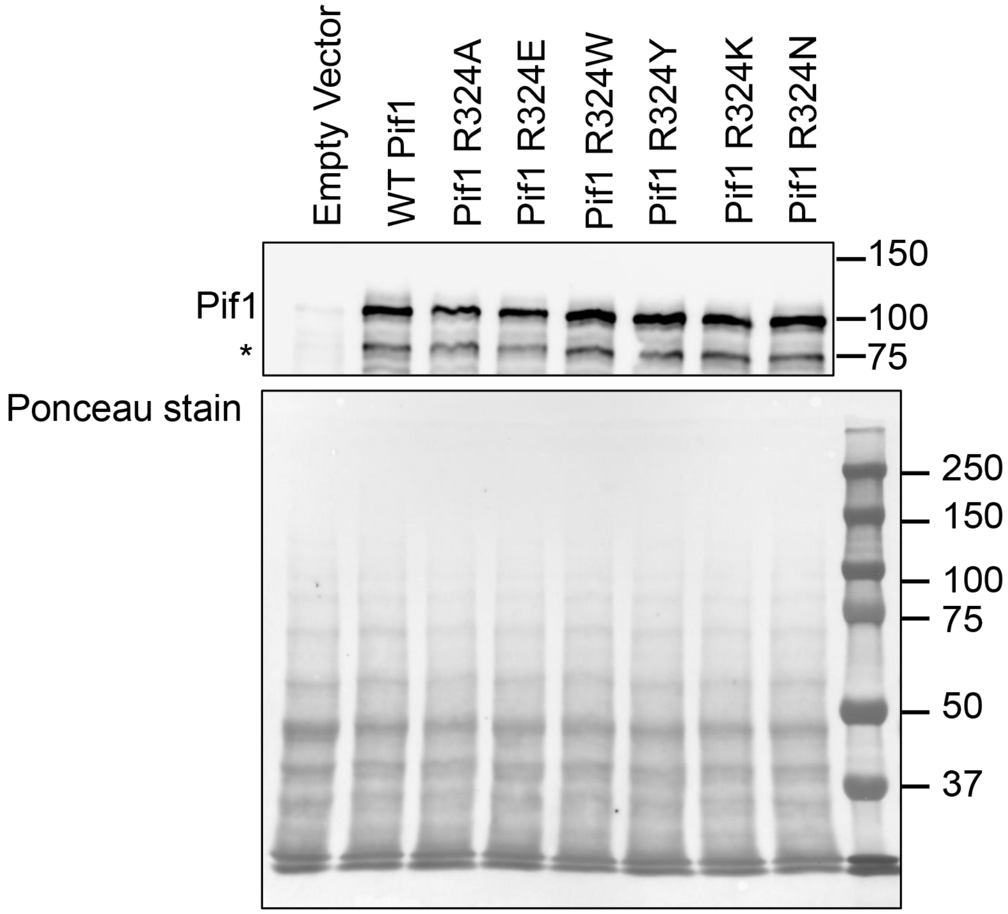

Supplementary Figure 9. Expression of ScPif1 variants *in vivo*

Western blot shows expression of each of the R324 ScPif1 variants. The band at about 100 kDa is the appropriate size for full length ScPif1. The band marked by an * is likely a Pif1 cleavage product. The Ponceau stained membrane shows equivalent protein loading in each lane. Source gels are provided as a Source Data file.

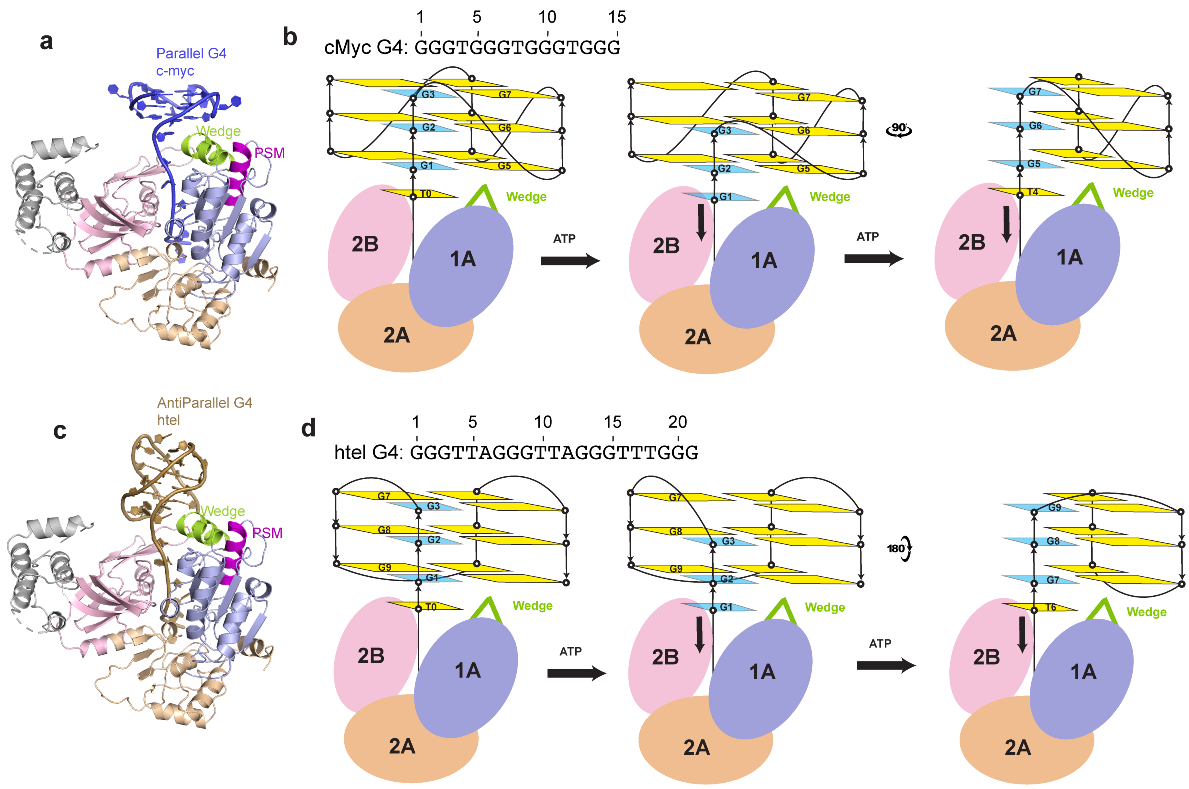

Supplementary Figure 10. Mechanisms of unfolding c-Myc and human telomere (htel) G4 DNA by ScPif1

(**a**) Model of ScPif1 in complex with c-Myc G4 DNA. (**b**) Proposed mechanism for unfolding c-Myc parallel G4 DNA involves the G3-triplex intermediate. (**c**) Model of ScPif1 in complex with htel G4 DNA (**d**) Proposed mechanism for unfolding htel antiparallel G4 DNA involves the G3-triplex intermediate.

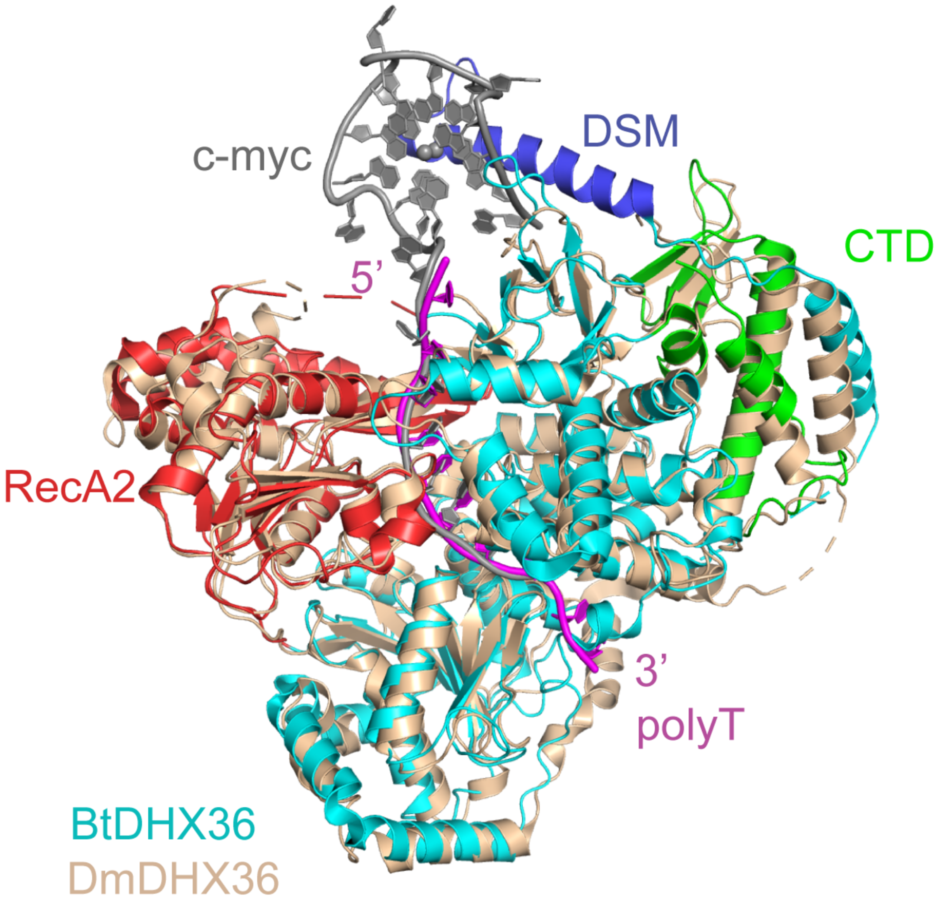

Supplementary Figure 11. Structural superposition of BtDHX36-G4 and with DmDHX36-ssDNA

The figure shows the BtDHX36-G4 (Bovine) (PDB code: 5VHE) and DmDHX36-ssDNA (Drosophila) (PDB code: 5N8S) structures in cartoon mode. BtDHX36 is colored in wheat and c-Myc G4 is colored in gray, with the DSM, RecA2, and CTD domains highlighted in blue, red, and lime, respectively. DmDHX36 is colored in cyan and ssDNA is colored in magenta.

Supplementary Table 1 | Oligonucleotides for crystallization, binding assay, unwinding and unfolding experiments

Substrate	Sequence
AT11	5’-**TGGTGGTGGTTGTTGTGGTGGTGGTGGT**-3’
T7-AT11	5′-TTTTTTT**TGGTGGTGGTTGTTGTGGTGGTGGTGGT**-3′
AT11-FAM	5′-**TGGTGGTGGTTGTTGTGGTGGTGGTGGT**/36FAM/-3′
T7-AT11-FAM	5′-TTTTTTT**TGGTGGTGGTTGTTGTGGTGGTGGTGGT**/36-FAM/-3′
T7-FAM	5′-TTTTTTT/36-FAM/-3′
Non-forked duplex	5′-/56-FAM/TTTTTTTTTTTTTTCGCTGATGTCGCCTGG-3′<br>5′-CCAGGCGACATCAGCG-3′
Forked duplex	5′-/56-FAM/TTTTTTTTTTTTTTCGCTGATGTCGCCTGG-3′<br>5′-CCAGGCGACATCAGCGTTTTTTTTTTTTTT-3′
Duplex trap	5′-CGCTGATGTCGCCTGG-3′
FAM-T16-AT11	5′-/56-FAM/TTTTTTTTTTTTTTTT**TGGTGGTGGTTGTTGTGGTGGTGGTGGT**TT-3′
AT11 trap	5′-CCACCACCACCACAACAACCACCACC-3′
AT11 C trap	5′-GGTGGTGGTTGTTGTGGTGGTGGTGG-3′
c-Myc	5′-/56-FAM/TTTTTTTTTTTTTTT**GAGGGTGGGTAGGGTGGG**TAA-3′
c-Myc trap	5′-CCCACCCTACCCACCC-3′
c-Myc C trap	5′-GGGTGGGTAGGGTGGG-3′
c-Myc reporter	5′-TTTTTTTTTTTTTTTGA**GGGTGGGTAGGGTGGG**TAACGCTGATGTCGC-3′<br>5′-/56-FAM/GCGACATCAGCGTTTTTTT-3′
Reporter trap	5′-GCGACATCAGCG-3′

Duplex forming sequences are underlined. G4 forming sequences are bold.

Supplementary Table 2 | PCR primers for construction of Pif1-mutant plasmids for *in vivo* experiments

Substrate	Sequence
R324A For	5′-GCGGATAAACTCTATAAAAAAGTTCGTGCTTCTCGAAAGCACCTAAGGCGCTG-3′
R324A Rev	5′-CAGCGCCTTAGGTGCTTTCGAGAAGCACGAACTTTTTTATAGAGTTTATCCGC-3′
R324E For	5′-GCGGATAAACTCTATAAAAAAGTTCGTGAATCTCGAAAGCACCTAAGGCGCTG-3′
R324E Rev	5′-CAGCGCCTTAGGTGCTTTCGAGATTCACGAACTTTTTTATAGAGTTTATCCGC-3′
R324K For	5′-GCGGATAAACTCTATAAAAAAGTTCGTAAGTCTCGAAAGCACCTAAGGCGCTG-3′
R324K Rev	5′-CAGCGCCTTAGGTGCTTTCGAGACTTACGAACTTTTTTATAGAGTTTATCCGC-3′
R324N For	5′-GCGGATAAACTCTATAAAAAAGTTCGTAACTCTCGAAAGCACCTAAGGCGCTG-3′
R324N Rev	5′-CAGCGCCTTAGGTGCTTTCGAGAGTTACGAACTTTTTTATAGAGTTTATCCGC-3′
R324W For	5′-GCGGATAAACTCTATAAAAAAGTTCGTTGGTCTCGAAAGCACCTAAGGCGCTG-3′
R324W Rev	5′-CAGCGCCTTAGGTGCTTTCGAGACCAACGAACTTTTTTATAGAGTTTATCCGC-3′
R324Y For	5′-GCGGATAAACTCTATAAAAAAGTTCGTTACTCTCGAAAGCACCTAAGGCGCTG-3′
R324Y Rev	5′-CAGCGCCTTAGGTGCTTTCGAGAGTAACGAACTTTTTTATAGAGTTTATCCGC-3′

Supplementary Table 3 | Plasmids used in this study

Name	Plasmid information	Reference
pMB13	pRS414 empty vector (*CEN ARES TRP1*)	ref[37]
pCG17	pRS414-PIF1 promoter-PIF1WT-3xFLAG	ref[37]
pCG18	pCG17-Pif1-K264A	ref[37]
REC0361	pCG17-Pif1-R324A	This study
REC0363	pCG17-Pif1-R324E	This study
REC0366	pCG17-Pif1-R324K	This study
REC0368	pCG17-Pif1-R324N	This study
REC0371	pCG17-Pif1-R324W	This study
REC0375	pCG17-Pif1-R324Y	This study

Supplementary Table 4 | Yeast strains used in this study

Name	Genotype	Reference
MBY77	*YPH500, hxt13:URA3, pif1::HIS3MX6*	ref[37]
YCG59	*W303 diploid, PIF1/pif1::NatMX6, DNA2/dna2::KanMX6*	ref[37]
G0339	*W303 haploid, pif1::NatMX6, dna2::KanMX6*	This study
G0587	*W303 haploid, pif1::NatMX6, DNA2WT, plasmid pMB13 (pRS414 vector)*	This study
G0591	*W303 haploid, pif1::NatMX6, DNA2WT, plasmid pCG17 (pCG17-PIF1WT-3xFLAG)*	This study
G0595	*W303 haploid, pif1::NatMX6, DNA2WT, plasmid pCG18 (pCG17-pif1K264A-3xFLAG)*	This study
G0599	*W303 haploid, pif1::NatMX6, DNA2WT, plasmid REC0361 (pCG17-pif1R324A-3xFLAG)*	This study
G0604	*W303 haploid, pif1::NatMX6, DNA2WT, plasmid REC0363 (pCG17-pif1R324E-3xFLAG)*	This study
G0608	*W303 haploid, pif1::NatMX6, DNA2WT, plasmid REC0371 (pCG17-pif1R324W-3xFLAG)*	This study
G0611	*W303 haploid, pif1::NatMX6, DNA2WT, plasmid REC0375 (pCG17-pif1R324Y-3xFLAG)*	This study
G0623	*W303 haploid, pif1::NatMX6, DNA2WT, plasmid REC0366 (pCG17-pif1R324K-3xFLAG)*	This study
G0627	*W303 haploid, pif1::NatMX6, DNA2WT, plasmid REC0368 (pCG17-pif1R324N-3xFLAG)*	This study
G0638	*YPH500, hxt13::URA3, pif1::HIS3MX6, plasmid pMB13 (pRS414 vector)*	This study
G0642	*YPH500, hxt13::URA3, pif1::HIS3MX6, plasmid pCG17 (pCG17-PIF1WT-3xFLAG)*	This study
G0646	YPH500, hxt13::URA3, pif1::HIS3MX6, plasmid pCG18 (pCG17-pif1K264A-3xFLAG)	This study
G0650	YPH500, hxt13::URA3, pif1::HIS3MX6, plasmid REC0361 (pCG17-pif1R324A-3xFLAG)	This study
G0654	YPH500, hxt13::URA3, pif1::HIS3MX6, plasmid REC0363 (pCG17-pif1R324E-3xFLAG)	This study
G0658	YPH500, hxt13::URA3, pif1::HIS3MX6, plasmid REC0366 (pCG17-pif1R324K-3xFLAG)	This study
G0662	YPH500, hxt13::URA3, pif1::HIS3MX6, plasmid REC0368 (pCG17-pif1R324N-3xFLAG)	This study
G0666	YPH500, hxt13::URA3, pif1::HIS3MX6, plasmid REC0371 (pCG17-pif1R324W-3xFLAG)	This study
G0670	YPH500, hxt13::URA3, pif1::HIS3MX6, plasmid REC0375 (pCG17-pif1R324Y-3xFLAG)	This study
